# Delta band activity contributes to the identification of command following in disorder of consciousness

**DOI:** 10.1038/s41598-021-95818-6

**Published:** 2021-08-11

**Authors:** Gonzalo Rivera-Lillo, Emmanuel A. Stamatakis, Tristan A. Bekinschtein, David K. Menon, Srivas Chennu

**Affiliations:** 1 Neuroscience Department, Faculty of Medicine, Universidad de Chile, Santiago, Chile; 2 Physical Therapy Department, Faculty of Medicine, Universidad de Chile, Santiago, Chile; 3 Research and Develop Unit, Los Coihues Clinic, Santiago, Chile; 4 Division of Anaesthesia, Department of Medicine, School of Clinical Medicine, University of Cambridge, Cambridge, UK; 5 Department of Clinical Neurosciences, Wolfson Brain Imaging Centre, School of Clinical Medicine, University of Cambridge, Cambridge, UK; 6 Cambridge Consciousness and Cognition Lab, Department of Psychology, University of Cambridge, Cambridge, CB2 3EB UK; 7 School of Computing, University of Kent, Medway, UK; 8 Department of Clinical Neurosciences, University of Cambridge, Cambridge, UK

**Keywords:** Neurological disorders, Diseases of the nervous system

## Abstract

The overt or covert ability to follow commands in patients with disorders of consciousness is considered a sign of awareness and has recently been defined as cortically mediated behaviour. Despite its clinical relevance, the brain signatures of the perceptual processing supporting command following have been elusive. This multimodal study investigates the temporal spectral pattern of electrical brain activity to identify features that differentiated healthy controls from patients both able and unable to follow commands. We combined evidence from behavioural assessment, functional neuroimaging during mental imagery and high-density electroencephalography collected during auditory prediction, from 21 patients and 10 controls. We used a penalised regression model to identify command following using features from electroencephalography. We identified seven well-defined spatiotemporal signatures in the delta, theta and alpha bands that together contribute to identify DoC subjects with and without the ability to follow command, and further distinguished these groups of patients from controls. A fine-grained analysis of these seven signatures enabled us to determine that increased delta modulation at the frontal sensors was the main feature in command following patients. In contrast, higher frequency theta and alpha modulations differentiated controls from both groups of patients. Our findings highlight a key role of spatiotemporally specific delta modulation in supporting cortically mediated behaviour including the ability to follow command. However, patients able to follow commands nevertheless have marked differences in brain activity in comparison with healthy volunteers.

## Introduction

A significant challenge in the clinical study of disorders of consciousness (DoC) is the measurement of residual cognitive functions, and the potential for the recovery thereof. In these patients, the overt or covert ability to follow commands is considered an unequivocal sign of awareness, which requires some degree of preserved cognitive function. Patients with unresponsive wakefulness syndrome (UWS, traditionally called vegetative state) are described as unable to engage with the sensory processing of external or internal stimuli and/or to produce voluntary motor activity^1^. On the other hand, patients in a minimally conscious state (MCS) show unequivocal evidence of interaction with the environment or themselves^2^. However, some MCS patients still cannot produce voluntary motor behaviour (MCS minus) while others can display voluntarily activity in response to different instructions (MCS plus)^3^. This ability to follow instructions involves the coordinated interaction of cortical networks to support high-level cognition, and it has recently been defined as cortically mediated behaviour^4^. This underscores the need to combine behavioural evidence with functional brain imaging data to understand the neural mechanisms that allow us to identify patients with the ability to follow commands (command-following or CF) and patients that cannot follow commands (non-command following or N-CF).

In recent years, mental imagery (where patients are asked to imagine motor activities in response to specific questions) measured with functional magnetic resonance imaging (fMRI) has become the research standard for establishing covert awareness in non-communicative brain-injured patients^5^. Furthermore, analysis of measures derived from electroencephalography (EEG) such as event-related potential (ERP)^6^, connectivity^7,8^, information theory^9^, and spectral markers^10^ have revealed their clinical relevance in providing an accurate diagnosis and for detecting such covert cognition. Most of these variables are extracted from spectral features of EEG signals in the delta, theta and alpha bands^11^.

Under a predictive coding framework^12^, ERP responses have been associated with successive levels of hierarchical predictive complexity^6,13,14^, and depend on organised, spatiotemporally specific modulations of the delta, theta, and alpha bands in response to auditory stimuli. Thus, the spatiotemporal dynamics of low frequency modulations in the EEG are thought to coordinate cortical activity across large-scale task-relevant networks^15,16^. Building on research in basic cognitive neuroscience, several studies have demonstrated oscillatory activity features in DoC patients^17–19^, but is not clear if differences in the spatiotemporal features of activity in different frequency bands can explain differences between CF and N-CF patients. Furthermore, the differences or similarities in spectral changes between healthy controls and DoC patients who can follow command remain unknown, either at the group level or at the single-subject level. To address this gap in knowledge, we combined evidence from fMRI mental imagery tasks and high-density EEG data collected during a hierarchical auditory prediction task, to identify the differences in spectral event-related modulation correlated with the ability to follow commands in DoC patients. We tested the hypothesis that low-frequency modulations in specific spatiotemporal windows can distinguish between healthy volunteers, CF and N-CF patients.

## Materials and methods

### Patients

The study was conducted in the Division of Anaesthetics at the University of Cambridge (Cambridge, UK). Patients included here were assessed during 4–5 days as part of a comprehensive clinical evaluation at Addenbrooke’s Hospital in Cambridge. This study was approved by the National Research Ethics Service for EEG measures (National Health Service, UK; LREC reference 99/391) in accordance with the declaration of Helsinki. Considering DoC patients are not able to provide informed consent, this was obtained from next of kin of patients prior to investigation. Data from a convenience sample of patients were assessed using high-density EEG, fMRI imagery and behavioural measures between March 2010 and March 2014 were included in this study. Repeated assessments of the same patients were included if at least six months had elapsed between assessments. In total, 24 datasets from 21 patients were included. Of the patients, 12 had a behavioural diagnosis of UWS, 8 MCS− and 4 MCS+, based on the highest score observed across repeated assessment with the Coma Recovery Scale-Revised during their admission. Diagnosis, time since injury, aetiology, gender, fMRI diagnosis and highest CRS-R score are shown in Table 1.

**Table 1:** Main features of DoC group.

		CRS-R diagnosis	CRS-R score	fMRI Diagnosis	Aetiology	Age (years)*	Months post-injury	Follow commands
1	Patient 1	UWS	8	Negative	Traumatic	47	48	N-CF
2	Patient 2	UWS	6	Negative	Traumatic	50	17	N-CF
3	Patient 3 (2nd ev.)	UWS	8	Negative	Traumatic	39	27	N-CF
4	Patient 4	UWS	7	Negative	Anoxic	40	14	N-CF
5	Patient 5	UWS	7	Negative	Anoxic	23	5	N-CF
6	Patient 5 (2nd ev.)	UWS	7	Negative	Anoxic	22	11	N-CF
7	Patient 6	UWS	7	Negative	Anoxic	72	7	N-CF
8	Patient 7	UWS	6	Negative	Anoxic	77	8	N-CF
9	Patient 8	UWS	7	Negative	Anoxic	19	8	N-CF
10	Patient 9	MCS-	11	Negative	Anoxic	21	46	N-CF
11	Patient 10	UWS	7	Negative	Anoxic	21	11	N-CF
12	Patient 11	MCS-	12	Negative	Traumatic	37	86	N-CF
13	Patient 12	MCS-	10	Negative	Traumatic	54	26	N-CF
14	Patient 13	MCS-	9	Negative	Anoxic	71	11	N-CF
15	Patient 14	MCS +	7	Negative	Traumatic	56	7	CF
16	Patient 15	UWS	7	Positive	Traumatic	36	8	CF
17	Patient 16	UWS	7	Positive	Traumatic	24	14	CF
18	Patient 11 (2nd ev.)	MCS +	10	Positive	Traumatic	30	78	CF
19	Patient 17	MCS-	9	Positive	Anoxic	29	6	CF
20	Patient 18	MCS +	18	Positive	Traumatic	39	11	CF
21	Patient 3 (1st ev.)	MCS-	8	Positive	Traumatic	37	15	CF
22	Patient 19	MCS-	9	Positive	Traumatic	26	5	CF
23	Patient 20	MCS-	7	Positive	Traumatic	20	19	CF
24	Patient 21	MCS +	13	Positive	Traumatic	23	4	CF

	Control subjects	Age (y)	Sex					
	Control 1	35	F					
	Control 2	29	F					
	Control 3	26	F					
	Control 4	25	F					
	Control 5	25	M					
	Control 6	25	M					
	Control 7	28	M					
	Control 8	33	M					
	Control 9	25	F					
	Control 10	30	M					

*At the moment of EEG recording.

### Healthy volunteers

To compare with healthy volunteers, we included previously reported EEG data from 10 neurologically healthy adults, collected in *attend-sequences* condition in a modified version of the global–local paradigm^12^. Healthy volunteers gave written informed consent and were paid £10/h for their time. Ethical approval was provided by the Cambridge Psychology Research Ethics Committee, in accordance with the declaration of Helsinki. Gender and age of healthy volunteers are shown in Table 1.

### fMRI data collection and interpretation

fMRI data were collected with the tennis and spatial navigation imagery task as described by Owen et al.^20^. Evidence of tennis and/or spatial navigation imagery was ascertained using the same statistical methodology as described there^20^. Briefly, for each scan, a general linear model contrasting periods of active imagery with rest periods was computed. Contrasts have been restricted to the brain locations within the supplementary motor area and the parahippocampal gyrus, as defined in the Harvard–Oxford Cortical Structural Atlas. A threshold was established with gaussian random-fields theory at a cluster-level z value of more than 2.3 (corrected P < 0.05), indicating the ability to follow commands. The regions of interest were transformed from standard space (according to the criteria of the Montreal Neurological Institute) to fit each subject’s structural image, using a method involving 12 degrees of freedom.

### Command-following and non-command-following classification

Patients were classified as CF if they provided positive evidence of the ability to follow commands, either behaviourally with a CRS-R classification of MCS plus, or by imagining playing tennis or spatial navigation imagery in the fMRI task. Patients were classified as N-CF if they had a behavioural classification of UWS or MCS minus, and a negative result in the fMRI mental imagery task.

### EEG data collection and experimental conditions

Data were acquired through a 128-channel high-density EEG, referenced to vertex and sampled at 250 Hz using the Net Amps 200 amplifier (Electrical Geodesics Inc., Oregon, USA). Data from patients were acquired under a clinical protocol where we ensured that the patient was aroused at the beginning of the recording session. EEG data were collected during the administration of the global–local paradigm^6^, described in detail in Chennu et al.^12^. In brief, stimuli corresponded to 2 kinds of tones (A and B) presented in 6 different sequences. Sequences were monaural or interaural. Data from monoaural sequences were used for analysis. A sequence comprised of a series of 5 complex sounds (lasting 50 ms in duration), each sound was composed of 3 sinusoidal waves (350 Hz, 700 Hz, or 1400 Hz for tone A, or 500 Hz, 1000 Hz, or 2000 Hz for tone B). These sequences were *locally* regular (hereafter local standard sequences), consisting of five identical tones (AAAAA or BBBBB), or *locally* irregular (hereafter local deviant sequences), consisting of four identical tones (AAAAB or BBBBA). These sequences were presented in blocks in which a *globally* regular sequence was presented 71.5% of the time (hereafter global standard sequences). The rest of the time, we presented a globally irregular sequence that was either also locally irregular (when the globally regular sequence in that block was also locally regular) or locally regular (when the globally regular sequence in that block was locally irregular) (hereafter global deviant sequences). The silent interval between consecutive sequences was randomly sampled from a uniform distribution between 700 and 1000 ms after the fifth tone. Each participant was presented with 10 blocks of such stimuli with breaks in between. Each block lasted ~ 4 min, with approximately 160 sequences in per block. Eight of these were experimental blocks, and 2 were control blocks where all types of sequences were equally probable.

### EEG preprocessing and data selection

EEG Preprocessing and data analysis were conducted using EEGLAB toolbox^21^, and the Fieldtrip toolbox^22^, using custom scripts built in Matlab® 2013b. Data were filtered between 0.1 and 20 Hz and epoched between − 300 and 1300 ms relative to the first tone in a sequence. Epochs containing excessive noise or muscular artefacts were rejected through a semi-automatic process. Outliers were defined as values larger than two standard deviations from the mean value for each channel, across all epochs. They were rejected or retained based on visual inspection. Rejected channels were interpolated using spherical spline interpolation^23^. Independent component analysis (ICA) was used to remove ocular artefacts and muscle noise sources. For ERP analysis, trials from each kind of stimuli were segmented in epochs from − 300 to 1300 ms relative to the onset of the first tone of each sequence and baseline corrected.

Because the global–local task has been designed to extract ERP responses, additional preprocessing was conducted to create longer epochs and extract frequency components from the data. To reduce boundary effects at the lower frequencies while preserving the frequency content, baseline data of each epoch were used to generate a 500-ms mirror padding^24^. Similarly, the last 200 ms of each epoch was used to add a mirror padding of the 500 ms at the end of the same epoch. As a result, the length of each epoch was from 0.8 s before the first tone in the sequence until 1.8 s after.

We calculated time–frequency representations between 1 and 13 Hz (delta 1–4 Hz, theta 4–8 Hz and alpha, 8–13 Hz) through Fourier analysis using a sliding Hanning window in 10-ms steps. Hanning window tapers the data fully to zero at the edge of the window, minimising edge artefacts. The length of the sliding windows was adapted as a function of frequency^24^, with longer time segments for the lower frequencies and shorter time segments for the higher frequencies. In this way, we considered 3 cycles for frequencies over 3 Hz and 1 cycle for frequencies under 3 Hz. The relative change of power spectrum after the onset of stimuli was calculated by event-related synchronisation (ERS) using the formula ERS% = (data − reference)/reference × 100^25^, where data corresponded to each time/frequency bin, and the reference is the average of time/frequency bins at 0.3 s before the first tone.

### Statistical analysis

#### ERP and cluster analysis

Event-related potential (ERP) analysis was conducted to determine significant differences between the stimuli related to local and global effects at specific time windows^12^. For the local effect, an early window between 0 and 0.3 s after the fifth tone was used to determine differences between local standard and local deviant sequences. For the global effect, a window between 0 and 0.65 s after the fifth tone was used to determine differences between global standard and global deviant sequences. A cluster-based permutation method, corrected by temporal adjacency and spatial neighbouring, was used to identify statistical differences between stimuli at specific temporal window and channels^26^. In brief, to evaluate the hypothesis that the experimental conditions produce different responses at the group level (standard versus deviant stimuli), we used non-parametric statistical testing to calculate the probability distribution obtained with the Monte Carlo method (permutation test)^26^. Separately for global and local effects, deviant and standard trials were grouped in one set and divided into two subsets through random partitioning. Channel-time sample estimates from both subsets were compared with a two-sided t-test. Then, we grouped all the samples whose t-value was larger than the critical alpha level (0.05) with a temporal and spatial adjacency criterion. The sum of all t-values in each cluster was saved, and only the clusters with the maximum and minimum values were conserved. We repeated the procedure 1000 times to generate the non-parametric null distribution and determined the p-value based on the proportion of the distribution, resulting in a larger test statistic than was observed in the original deviant and standard trials (separately for global and local effects). This implies that both large negative and large positive T-statistics are selected for later clustering. If this non-parametric cluster-level p-value was smaller than the critical alpha-level (0.05), we rejected the null hypothesis and accepted the alternative hypothesis that the data were significantly different and came from different distributions.

These spatiotemporal cluster were used to calculate the Pearson’s linear correlation coefficients between the ERS at the frequency of interest and the two main grand—average ERP responses observed across the average of all stimuli at 300 ms after the first tone and 300 ms after the fifth tone. Similarly, linear correlation coefficients have been estimated between ERS at the frequency of interest and ERP related to local and global effects. False discovery rate correction (FDR) was applied.

Considering that in some DoC patients is not possible to identify the peak amplitude of the ERP, we estimated the area under the curve (AUC) of the time-domain features at the temporal window related to the N1 component (− 0.6 to 0.3 s), the MMN (0–0.3 s) and the P3b (0.3–0.7 s). AUC was calculated using the trapezoidal method.

### Multinomial model

A 3-category regularised multinomial logistic regression model was implemented to identify a small number of important predictors that discriminate between CF, N-CF and the control group using the spectral variables (delta, theta and alpha band) at the time windows and clusters channels previously described. Also, we added as predictors to the model the average ERS in each band from all channels at the same temporal windows to reduce circularity risk. The averages of the ERS for each band and for each time window and clusters channels were used as variables to build the model. Thus, the combination of variables listed in Table 2 allowed us to build a model with 27 predictors. Variables were scaled by subtracting the mean and dividing by the standard deviation. Because low-frequency spectral variables are highly correlated, we employed an elastic net penalised approach using the glmnet package for R^27^. The algorithm implemented in the glmnet package fits the multinomial logistic model using regularised maximum likelihood^27^. The combination of ridge regression and lasso penalty implemented by the elastic net regularisation was tuned with an alpha value of 0.9 to obtain a predominant lasso penalty and reduce the number of predictors in the regression model. The degree of penalisation was selected by considering the largest λ value within 1 standard error (1SE) of the optimal model estimation based on leave-one-participant-out cross-validation. The Multinomial Deviance (MD) based on leave-one-out cross-validation allows select λ to optimize goodness of fit. MD is a goodness-of-fit statistic used when model-fitting is estimated by means of maximum likelihood. Lambda is a regularization parameter. As lambda increases, the number of nonzero components of coefficients decreases. The method leaves out a subject from the sample, fit the model for one hundred different λ values, and computed the goodness-of-fit error for each λ value. This computation is repeated 34 times to obtain 34 estimates of goodness-of-fit error at each λ value, where the mean and SD were calculated. The λ value corresponding to where the MD was smallest, defined the optimal model in terms of cross-validation. Our final model was chosen considering the largest λ at which the MD is within one standard error (1SE) of the smallest MD. Based on 1SE λ value, we estimated the ability of the optimal penalised multinomial logistic regression to classify participants accurately using the selected variables. Thus, if variables are helpful in identifying CF, NCF and control subjects, a more detailed analysis of variance of these variables should be necessary to explore event-related spectral differences across groups.

**Table 2:** Variables used to build the multinomial model.

Time windows	Cluster channels	Spectral band
1st–5th tone window (− 600 − 0 ms)	Frontal cluster	Delta
Early window (0–300 ms)	Parietal cluster	Theta
Late window (300–700 ms)	All channels	Alpha

Because to ERS and ERP are related to similar neurophysiological mechanisms, we computed the same regularised multinomial model to explore if the time-domain features related to ERP are helpful to distinguish between CF, NCF and control subjects. We included event-related amplitude modulation across temporal windows related to N1 (− 0.6 to 0.3 s, from all stimuli), MMN (0–0.3 s, from local deviant stimuli) and P3b components (0.3–0.7 s, from global deviant stimuli). N1 and MMN time-domain features were collected from the frontal cluster and P3b from the parietal cluster. Considering brain deformities in DoC subjects, we included the time-domain features from all channels at the same temporal windows and stimuli.

We estimated sampling distribution to compute confidence intervals based on bootstrapping. We generated 1000 bootstrap samples with replacement. Each bootstrap sample was used to calculate the regularised multinomial logistic regression model. In this way, we obtained two-sided confidence intervals (2.5–97.5 percentile) for the mean values in the three-class confusion matrix. The chance level was based on the probability of each outcome in the model, considering the sample size of each group (14/34; 10/34; 10/34), leading to a chance level of 41.1%, 29.4% and 29.4% for NCF, CF and control, respectively.

### Analysis of variance

For ERP, a one-way ANOVA was employed to compare AUC means between the three groups. For the ERS, an ANOVA was calculated to explore specific differences across groups and to visualize individual variability. The between-participant factors were groups (CF, N-CF and control) and the within-participant factors were the average of ERS across all stimuli for the spatiotemporal variables selected by the regularised multinomial logistic regression. Following a significant interaction, to determine the difference between groups at each level of each factor, we estimated simple main effects to identify the exact nature of the interaction using the error term y degree of freedom from the whole design. If the simple main effect was significant, we conducted post hoc analyses using t-tests and false discovery rate (FDR) to control for multiple comparisons. Mauchly’s test of sphericity was used to test the null hypothesis that the variances of the differences are equal, and Greenhouse–Geisser correction was applied if necessary^28^.

## Results

### Classification as command-following and non-command-following

Patients were classified as CF (Command Following) if they provided positive evidence of the ability to follow commands, either behaviourally with a CRS-R classification of MCS plus, or by imagining playing tennis or spatial navigation imagery in the fMRI task. Patients were classified as N-CF (Non-Command Following) if they had a behavioural classification of UWS or MCS minus, and a negative result in the fMRI mental imagery task.

Twenty-four datasets from 21 DoC patients were categorised based on their ability to follow commands. Patients with a positive indication in at least one of these measures was considered to be a command-following (CF). Patients with no response in either modality were considered to be non-command following (N-CF). Based on these criteria, 10 patients were identified as CF, and 14 were classified as N-CF.

**Figure 1:**
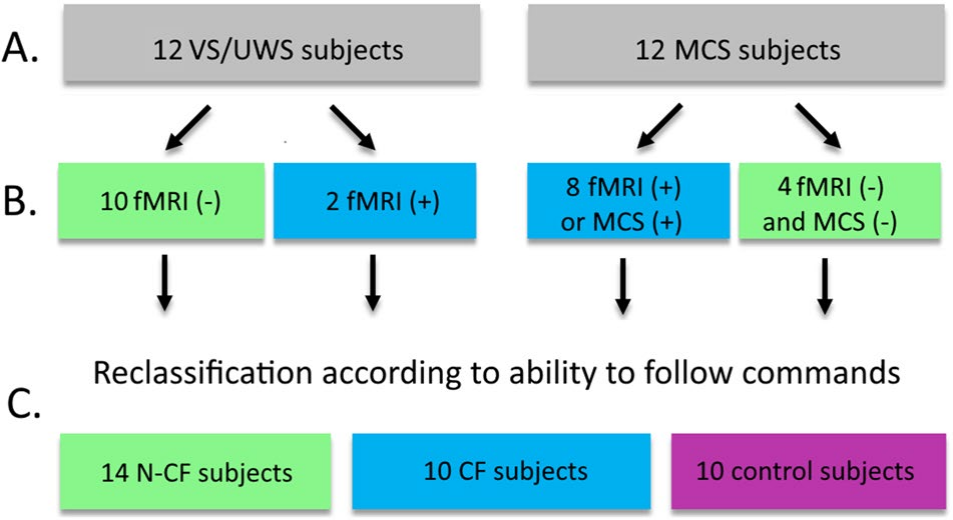
Classification of subjects. (**A**) Behavioural classification based on consecutive applications of the CRS-R. (**B**) The ability to follow commands is determined based on the evidence coming from fMRI imagery task and/or from CRS-R. (**C**) Reclassification of subjects according to their ability to follow commands.

Further, based on the above classification scheme, 2 of the 13 patients previously classified as UWS solely on their behavioural diagnosis were classified as CF, because they had positive evidence in the fMRI imagery task, thereby displaying covert cognition. Conversely, 3 behaviourally MCS patients were classified as N-CF due to a lack of evidence of command following in both the CRS-R and the fMRI imagery task. Figure 1 presents the classifications of the DoC patients.

### Low-frequency modulation was highly correlated to the ERP responses at different spatiotemporal clusters

**Figure 2:**
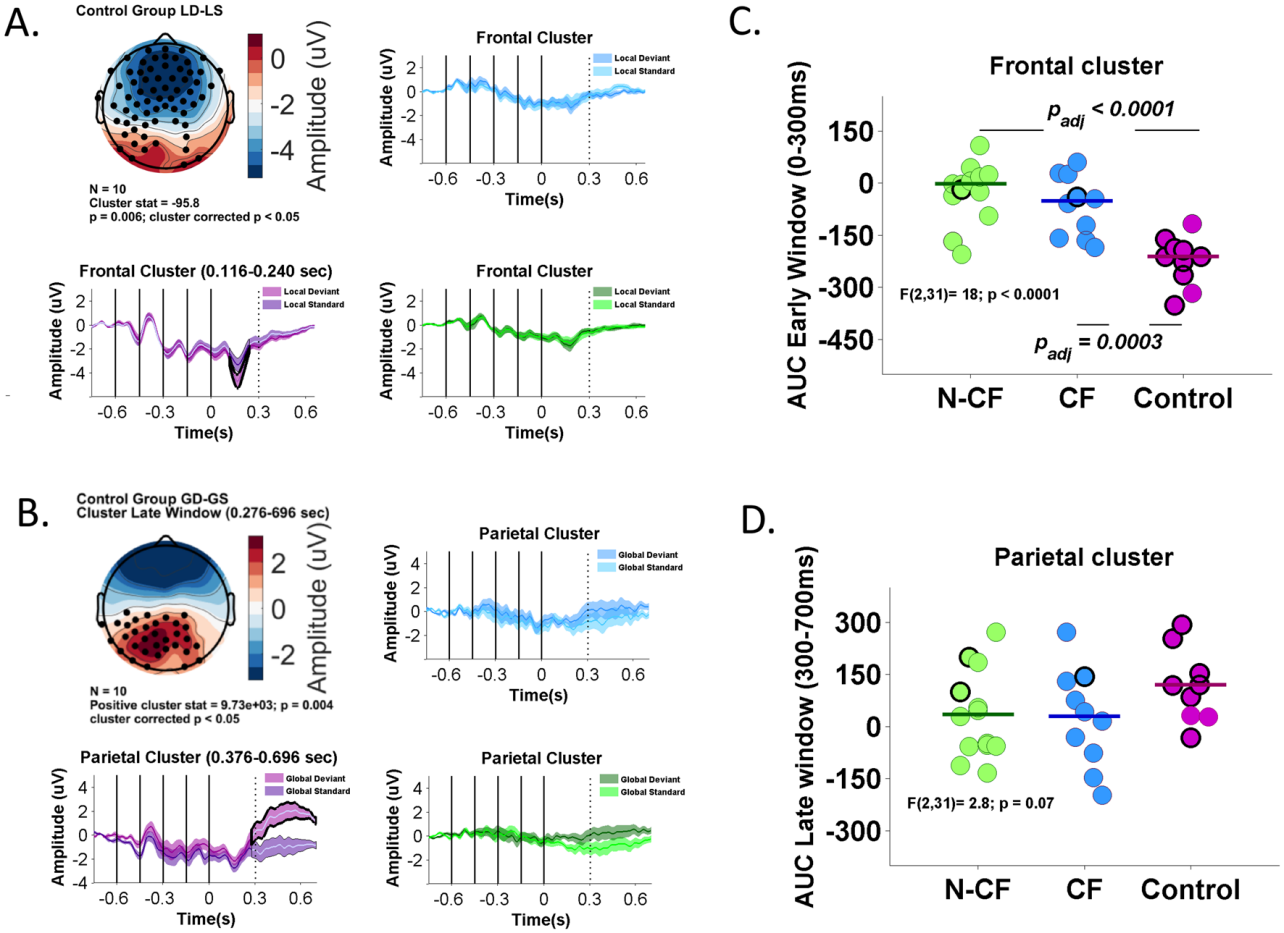
ERP responses at single subject and group level. (**A**) Spatial topography and channels (black dots) indicate where there are significant differences between stimuli related to the local effect (negative cluster stat). Plots shows grand-average ERP time courses for the local deviant and local standard stimuli at this cluster channels. Healthy subject (purple), CF (Blue), N-CF subjects (green). (**B**) Spatial topography and channels (black dots) indicate where there are significant differences between stimuli related to the global effect (positive cluster stat). Plots shows grand-average ERP time courses for the global deviant and global standard stimuli at this cluster channels. (**C**) Individual patient value for the local effect are shown in the scatter plot. Group-wise mean of the area under a curve (AUC) is indicated by horizontal line. circles with black edge shows significant differences at single-subject level. (**D**) Individual patient value for the global effect.

We conducted cluster-based permutation tests on ERP responses in each group at single subject-level. Similar to previous reports^12^, in the healthy controls, we confirmed differences for contrasts between stimuli related to local effect in an early time window after the fifth tone (0–300 ms, mismatch negativity MMN) at frontal cluster channels (Fig. 2A). Furthermore, in the late time window after the fifth tone, we confirmed differences for contrasts between stimuli related to global effect (300–600 ms, P3b) at parietal cluster channels (Fig. 2B). In contrast to healthy controls, DoC patients as a group showed no differences for contrasts between stimuli related to local and global effects (Fig. 2C, D).

**Figure 3:**
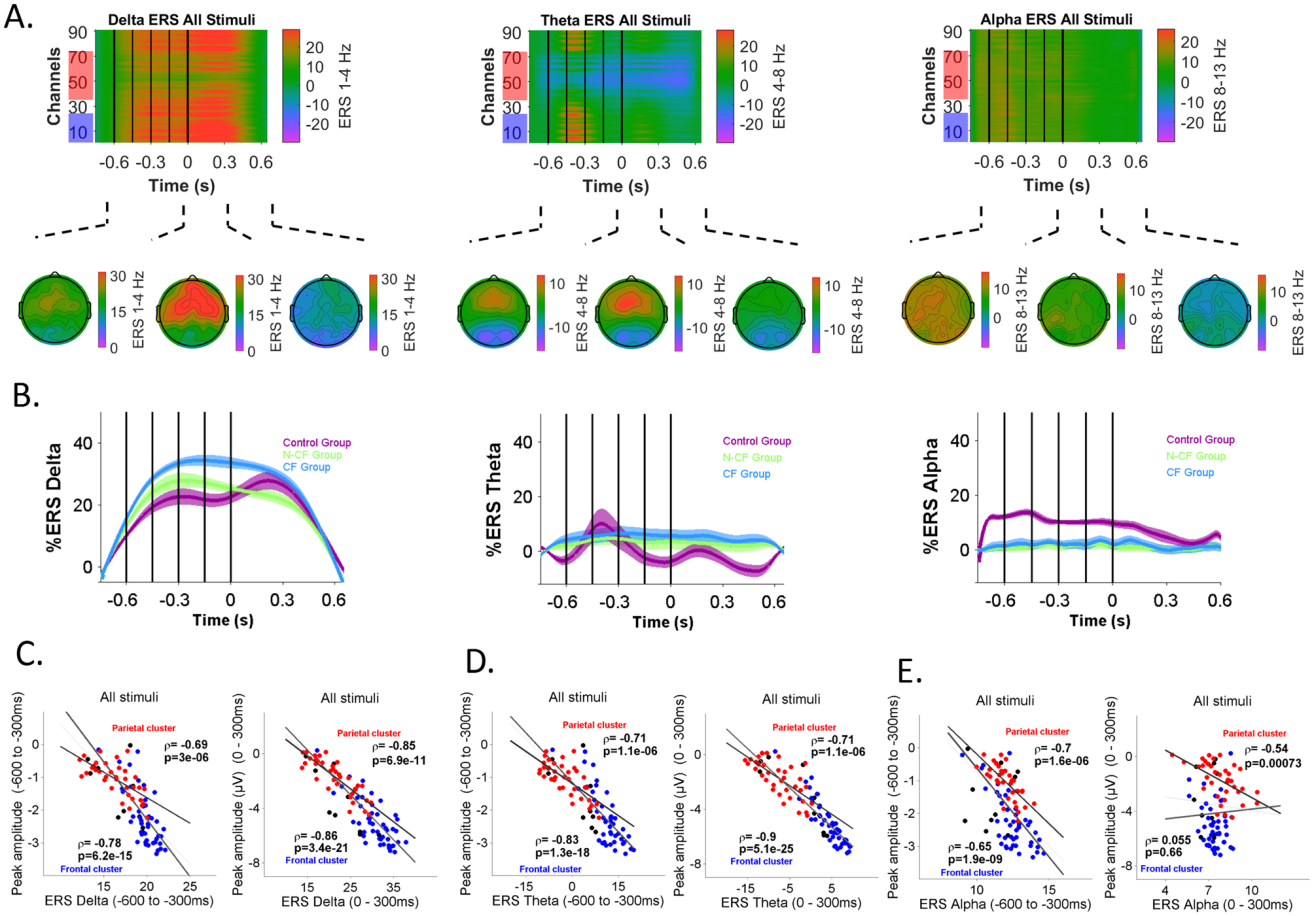
The temporospatial pattern of low-frequency modulation. (**A**) Relative changes in the time–frequency ERS delta (left), theta (middle) and alpha activity (right) related to the average of all stimuli for healthy subjects. Y axis display EEG channels, red box show parietal channels and blue box frontal channels. (**B**) Average of relative changes across CF (blue), N-CF (green) and the control group (purple) for all stimuli and all channels. (**C**)–(**E**) Correlation analysis for the frontal (blue) and parietal (red) cluster between the ERS at the frequency of interest and the two main ERP responses observed across the average of all stimuli.

At the same time, spectral modulation in the delta, theta, and alpha bands was estimated across all stimuli. In the healthy controls there was a predominant increase of activity at the delta and theta bands before presenting the fifth tone (time 0) and 300 ms afterwards (Fig. 3A). At these time windows, the activity had a predominant frontal spatial distribution over the scalp. Figure 3B plots the pattern of the spectral modulation averaged across all channels for CF (blue trace), N-CF (green trace) and the control group (purple trace) for each band of interest. Visually, CF and N-CF patients showed differences in delta band modulation (Fig. 3B left), whereas theta and alpha bands showed differences between patients and controls (Fig. 3B middle and right).

Then, in the control group, we estimated the correlation between the peak amplitude and the ERS when all stimuli in the task were considered. Figure 3C, D and E shows the correlation for the frontal and parietal cluster between the ERS at the frequency of interest and the two main ERP responses observed across the average of all stimuli at 300 ms after the first tone and 300 ms after the fifth tone. High correlation coefficients were observed for most of the frequency bands at parietal and frontal clusters (from rho =  −0.85, p adj. = 2.1e−10, to rho =  −0.54, p adj. = 0.0008) except for the alpha band in the frontal/early spatiotemporal cluster (rho = 0.055, p adj. = 0.69). At the same time, high correlations coefficients were observed between ERS at the frequency of interest and the ERP related to local and global effect (see supplementary Fig. 1). The high correlation coefficients between the main ERP markers used to detect perceptual processing and frequency modulation support the relevance of exploring cognitive abilities by describing modulation at frequencies of interest as an alternative or complementing the information provided by the ERP analysis or other EEG variables.

### Low-frequency spectral variables allow us to distinguish between CF, N-CF and healthy controls

**Figure 4:**
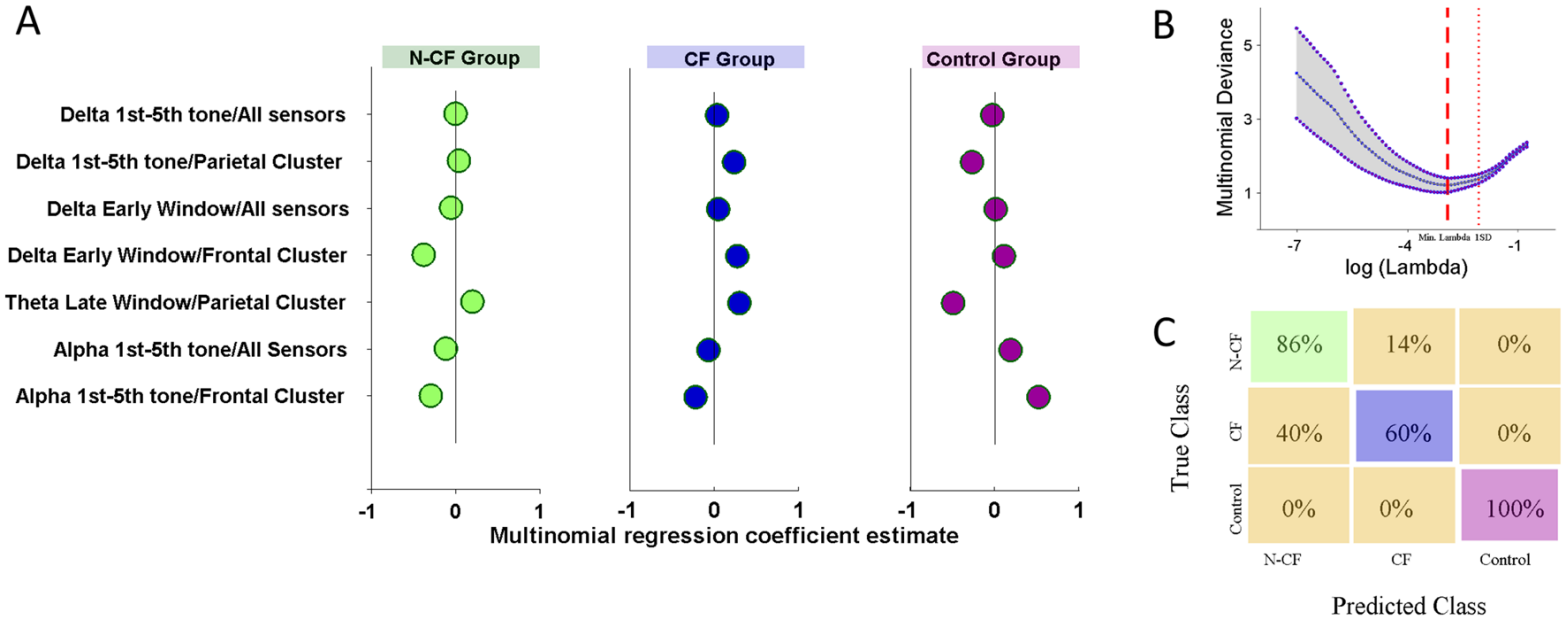
Coefficient estimates for a penalised multinomial logistic regression. (**A**) Spatiotemporal variables and their coefficients selected from the regularised multinomial model. (**B**) The method leaves out a subject from the sample, fits the model for 100 different λ values, and computes the goodness-of-fit error for each λ value. Red lines show the optimal degree of penalisation (lambda) related to the minimum value of multinomial deviance and one standard deviation. (**C**) Confusion matrix showing the percentage of predicted and true classes estimated by multinomial regression coefficients across the 3 groups.

The frontal and parietal clusters channels identified above were used to compute a penalised three-category multinomial logistic regression using elastic net regularisation to identify the most important spectral variables for discriminating between CF, N-CF and controls. These variables include measures of spectral modulation at various latencies of perceptual processing, indirectly indexing its depth. Thus, we used as predictors average ERS at delta, theta and alpha bands between 1st and 5th tones (− 0.6 to 0 ms), during the early (0–300 ms), and during the late (300–700 ms) time window (eighteen spatiotemporal spectral variables). Also, we added as variables to the model the average ERS in each band from all channels at the same time windows (1st–5th tone, early and late windows, adding nine variables) (Table 2). Thus, we built a model with 27 variables, and the subject group (CF, N-CF and control). Figure 4A summarises the regression coefficients of the seven variables selected by the regularised model. The optimal penalised multinomial model (Fig. 4B) was able to classify 28/34 subjects (~ 82%, 95% CI 76–100) accurately. Based on these seven spectral variables the model correctly classified 12/14 N-CF subjects (~ 86%, 95% CI 71–100), 6/10 CF subjects (60%, 95% CI 30–100) and 10/10 control subjects (100%, 95% CI 100–100). However, it is important to note that 40% (CI 0–70%) of the CF subjects are still classified as N-CF. The confusion matrix is displayed in Fig. 4C. Visually is possible to observe that modulation at frontal alpha during the 1st–5th tone window and parietal theta band at the late window are important variables to separate between DoC and control volunteers. In contrast, frontal delta modulation in the early window is an important discriminator of CF and N-CF subjects.

Because ERS and ERP are related to similar neurophysiological mechanisms and could lead to similar classification results, we explore whether the ERS variables’ contribution to detecting CF patients is different from time-domain variables related to ERP. We included the main time-domain features from the global–local paradigm to build the multinomial regression model with the same three classes (NCF, CF and control). Using the AUC ERP analysis proposed in Fig. 2, we included event-related amplitude modulation across the N1 temporal window (− 0.6 to 0.3 s, from all stimuli), MMN (0–0.3 s, from local deviant stimuli) and P3b (0.3–0.7 s, from global deviant stimuli). N1 and MMN time-domain features were collected from the frontal cluster and P3b from the parietal cluster. Considering brain deformities in DoC subjects, we included the time-domain features from all channels at the same temporal windows and stimuli. Under the same parameters described previously to estimate the multinomial model, the variables selected were the time-domain features from the MMN temporal window from the frontal cluster and all channels. This selection is similar to the event-related spectral variables selected previously. The new penalised multinomial model based on ERP variables correctly classified 9/10 control subjects (90%, 95% CI 70–100%) and 14/14 NCF subjects (100%, 95% CI 79–100%) (see supplementary Fig. 2). All CF subjects were misclassified as N-CF subjects (8/10) or healthy control (2/10). Similarly, when the seven ERS variables are used to classify subjects related to the behavioural label (VS/UWS, MCS, Control), we were able to identify 9/12 VS/UWS, 5/12 MCS and 10/10 control subjects. Together, these results suggest that ERS variables can complement ERP analysis to identify preserved cognitive functions in subjects with the ability to follow commands.

### The Delta band is the main variable to distinguish between CF and N-CF DoC participants

A more detailed analysis of the spatiotemporal variables selected by the regularised model was conducted to establish how the temporal dynamics of modulation at the selected bands contribute to perceptual processing in patient groups and to visualize the individual variability. We compared the mean of the frequency modulation across groups by means of a mixed ANOVA considering the groups as the between subjects factor (CF, N-CF and control) and the 7 variables selected in the regularised model as the within subject factor. A two-way interaction between spatiotemporal variables and groups confirmed the results showed in the penalised model (F(6,186) = 12.07, *p* < *0.0*001, partial η^2^ = 0.438).

**Figure 5:**
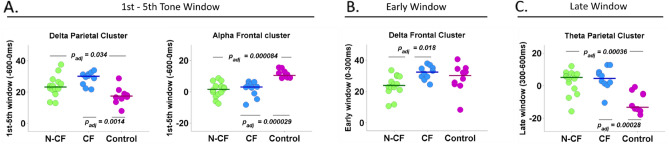
Mean contrast by group for the main spectral space/time variables selected for regularised multinomial regression. (**A**) ERS differences for the delta and alpha bands at parietal and frontal space cluster during the 1st–5th tone window. (**B**) ERS differences for the delta band at the frontal/early window. (**C**) ERS differences for the theta band at parietal/late window. Green dots display N-CF subjects, blue CF subjects, and purple control subjects. All p-value are corrected for multiple comparisons.

Figure 5 plots pairwise comparisons and individual values for the parietal and frontal spatiotemporal window variables for each band of interest. Delta and alpha band modulation showed differences between control and both DoC groups in the temporal window between the 1st and 5th tones at the parietal and frontal cluster respectively (Fig. 5A). Interestingly, delta modulation during the early window at the frontal cluster showed differences between DoC participants, with an increased modulation in CF subjects compared with N-CF subjects (Fig. 5B). This difference is still present in the early window when all channels were considered (early/all channels t(31) = 2.98, *p* adj. = 0*.*012). Similar to the alpha band, theta band modulation showed differences between control and both DoC groups during the late temporal window at the parietal cluster (Fig. 5C). Together, a more fine-grained analysis of the spectral modulation at the spatiotemporal variables selected in the multinomial model shows that the frontal/early window allow us to distinguish between DoC groups, with an increased modulation for the CF subjects in comparison with N-CF subjects. On the other hand, modulation at the parietal/delta and frontal/alpha during the 1st–5th tone and at the theta parietal/late spatiotemporal windows show a limit in the perceptual processing between control and both DoC groups. In this way, variables related to spectral changes at specific spatiotemporal windows used in the ANOVA and the multinomial model allow us to identify at group level and classify at individual level differences across NCF, CF and control subjects. In contrast, ERP responses showed differences only between DoC groups and controls (Fig. 2).

## Discussion

This study has described how variables related to event-related spectral changes at different bands can contribute to understanding the differences in perceptual processing and to identify DoC patients with and without the ability to follow commands. Despite the well-reported changes in the EEG neural activity between control and DoC subjects, a more detailed analysis of the spectral differences between DoC participants with and without cortically mediated behaviours allows us to explore key differences related to cognitive abilities. Differences between NCF and CF participants related to the modulation of the neural activity at specific spatiotemporal windows only could be statistically detected when the spectral variables are used, whereas the time-domain analysis at the same spatiotemporal windows did not show differences between DoC groups. Thus, whereas ERP and low-frequency spectral modulations could be related to similar neurophysiological processes, event-related spectral changes seem to be more robust to detect cognitive functions in the DoC subjects. These results underline the role of spectral modulations in perceptual prediction, and its clinical relevance for differentiating patients with higher-order cognitive function.

Previous EEG reports using the same cognitive task manipulating expectation and attention showed a P3b (indexing a global effect) as a specific marker of consciousness in DoC patients^13,14^. Our results show a low number of subjects where it was possible to identify a P3b component. This finding is according to evidence of previous reports in a large cohort of patients showing low sensitivity of this marker compared to other EEG variables^11^. In addition, the presence of a global effect to predict behaviourally overt consciousness recovery has been reported to have high specificity (~ 84%) and a high positive predictive value (80%) but with low sensitivity (35%) and low negative predictive value (42%)^29^.

Increased power in delta band, along with a reduction of power in the alpha band, is a replicated finding in DoC subjects^11,30^. However, at the same time, it has been proposed that modulatory inputs at the delta band have a key role in active control of neuronal excitability of cortical local ensemble, mainly during the rhythmic-mode processing task, tuning the excitability of the cortical local ensemble^15, 16,31^. The intrinsic rhythmic structure of the global–local paradigm allows us to explore the activity of modulatory delta band as a key feature across DoC subjects. Moreover, patterns of endogenous delta activity in the prefrontal cortex during predictive context tasks have a strong correlate in the top-down control driving posterior alpha activity^32^. Indeed, it has recently been showed that functional connectivity in delta-theta band is higher in patients being in a minimally conscious state than in those being in UWS^33^. Thus, the characteristically increased levels of spectral power at the delta band in DoC subjects should be analysed complementary with the endogenous and stimulus-related modulation as a mechanism contributing to the organisation of neural activity across other frequency bands.

Independently of their ability to follow commands, both groups of DoC subjects show similar features at the theta and alpha bands reflecting a marked border with the healthy subjects. This finding is consistent with evidence that shows the increment and decrement in the alpha band activity to route information to task relevant posterior cortices under top-down control^34^. Likewise, the theta band has been associated with various mechanisms of cognitive control^35^ necessary to maintain sensory evidence and to make predictions at different hierarchical temporal scales through working memory networks^36,37^. Further, changes at the theta band has been reported previously as a useful variable for identifying residual cognitive abilities in DoC groups^17^. Taken together, the temporal dynamics of the variables selected for the multinomial model showed an increment modulation at the delta band during the 1st to the 5th tone time window, accompanied by a modulation for the alpha band only in the control group. Later, in the early time window after the fifth tone, the CF and control group increased modulation at the delta band, but only the control group had changes in theta band in the late time window. These results are in concordance with previous reports, where at resting state the spectral power in the delta band is able to distinguish MCS minus from MCS plus^7^. Similarly, the power in the theta and alpha bands are effective at distinguishing UWS from other patient groups^11^, and alpha network metrics can distinguish between UWS, MCS minus and MCS plus^7^.

### Strength and limitations

Our study has several limitations. The richness of these data also makes it difficult to explore them in a large cohort of subjects, and this creates limitations in the methods of analysis. Thus, these results have the potential for clinical application, pending validation in a larger cohort where we confirm test out-of-sample generalisation accuracy. However, our complex approach to the analysis was combined with simpler approximations in the same data set, yielding similar results. Second, the EEG data was collected under an experimental task designed mainly to the analysis of ERP. However, this task has been used in a previous relevant work^10,11^ to conduct a comprehensive analysis of a large number of EEG variables, including connectivity, complexity and mutual information, entropy and spectral power. Third, the statistical analysis conducted does not allow a causal interpretation of our finding to explain the presence of cortically mediated behaviours in DoC subjects. At this point, is important to note the main issue with respect to DOC patients is that currently it is not possible to assert that a patient does not have cortically-mediated behaviour. Finally, despite the clinical protocol considering repeated assessments here, we were able to report the higher CRS-R score. Knowing the CRS-R subscores and the number of assessments is necessary to estimate the expected classification error. However, as we mentioned previously the clinical protocol considered several assessments reducing the expected error classification^38,39^.

Nevertheless, our study has several strengths. Data from serial behavioural assessment, functional brain imaging and high-density EEG allow us to explore in depth the remnant cognitive processes^40^. Moreover, combining these data allow us to detect cortically mediated behaviours to obtain a more precise classification of DoC subjects, doing more confident our results.

## Conclusion

Our study identified a group of variables that help to understand the differences in the perceptual processing in DoC subjects with and without evidence of the ability to follow commands. These results complement previous works showing the clinical relevance of the spectral variables related to ongoing activity or resting state to identify UWS/VS, MCS+ and MCS−^10,11^. Our results showed that a group of spectral spatiotemporal variables collected under the predictive context task could be useful for understanding differences in the neural mechanisms related to the preservation of cognitive capacities in DoC subjects. More important, however, is that it gives us valuable insight into the timing of spectral modulation needed to coordinate neural activity across task-relevant large-scale networks in CF and N-CF groups of DoC subjects. This can help to explain the preserved mechanism of the neural activity that supports remnant cognitive capabilities.

## Supplementary Information

Supplementary Figures.

## Data Availability

The datasets generated during and/or analyzed during the current study are available from the corresponding author on reasonable request.
