# Supplementary material for: Delta band activity contributes to the identification of command following in disorder of consciousness
Source: Sci Rep. 2021 Aug 11;11:16267. doi: 10.1038/s41598-021-95818-6 (PMC8357781; doi:10.1038/s41598-021-95818-6)
Supplement: Supplementary file 1 — Supplementary Figures. [file 41598_2021_95818_MOESM1_ESM.pdf]

Delta band activity contributes to identifying command-following in disorders of consciousness.

Gonzalo Rivera-Lillo, PhD.[1], Emmanuel A Stamatakis, PhD[2], Tristan A Bekinschtein, PhD [3], David K Menon, MD PhD. [2], Srivas Chennu PhD.[4]

Supplementary Information

Supplementary Figures

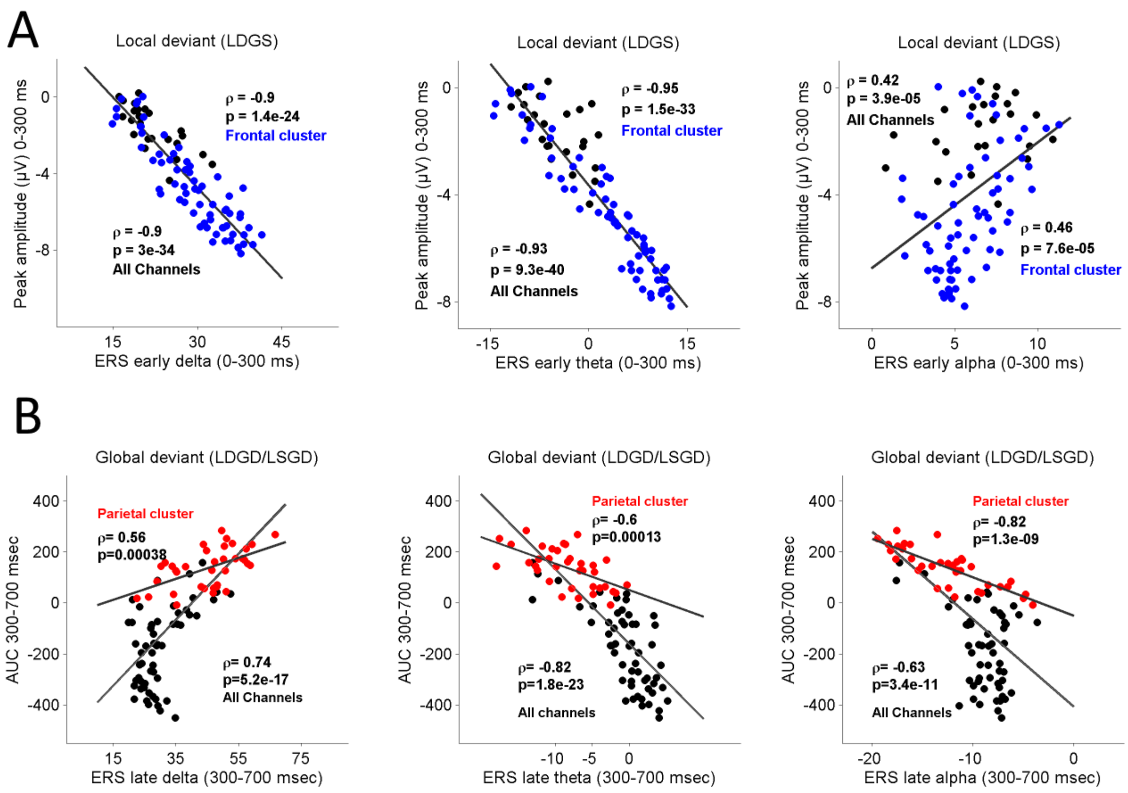

Supplementary Figure 1. Correlation for the frontal cluster (blue), parietal cluster (red) and the others channels (black) between the ERS at the frequency of interest and the two main ERP responses related to local (A) and global effect (B) in the healthy group.

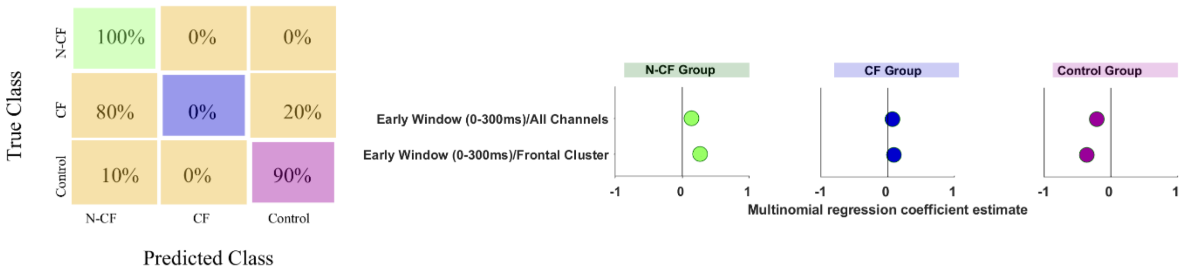

Supplementary Figure 2. Coefficient estimates for a penalised multinomial logistic regression using ERP variables. Left, confusion matrix showing the percentage of predicted and true classes estimated by multinomial regression coefficients across the 3 groups. Right, spatiotemporal variables and their coefficients selected from the regularised multinomial model.
